# Title: Haematological disorders revealing a raticide suicide attempt: A case report

**DOI:** 10.1192/j.eurpsy.2021.2186

**Published:** 2021-08-13

**Authors:** N. Messedi, W. Abid, I. Krichen, I. Frikha, R. Ouali, N. Halouani, C. Kallel, J. Aloulou

**Affiliations:** 1 Psychiatry (b), Hedi Chaker University hospital, sfax, Tunisia; 2 Hematology Laboratory, Habib Bourghiba University Hospital, Sfax, Tunisia; 3 Hematology Department, Hedi Chaker University Hospital, sfax, Tunisia

**Keywords:** Suicide, schizophrénia, rodenticides, coagulopathy

## Abstract

**Introduction:**

Suicide attempts are common in individuals with schizophrenia. These actions are marked by a greater lethality, due to the use of more violent means in particular the intentional ingestion of rodenticides.

**Objectives:**

To describe the gravity of the heamatological disorders revealing suicide attempts by a rodenticides in patient with schizophrénia.

**Methods:**

We repport the case of a patient who present a haematological disorders after an rodenticide intoxication.

**Results:**

A 41-year-old man with schizophrénia since 2011 was brought to the department of psychiatry in july 2020 for behavioral disorders. On arrival, the patient was oriented but reticent and refuse to tell his full story. On examination, his vital signs were normals, and he did not show any externalized bleeding. Bilogical tests revealed the prothrombin time (PT) was <10% with an isolated and unexplained fall in vitamin K-factors. The etiological investigation was negative. Later,the patient admitted attempted suicide by taken 4 rodenticide packages orally three days prior admission to hospital. The initial treatment with intravenous vitamin K almost daily is effective. An improvement in PT (35%) and vitamin K-dependent factors was observed after one week of treatment. A Normalization of hemostasis disorders was obtained after two weeks of treatment.

**Conclusions:**

It is imperative to suspect rodenticide intoxication in patient with scizophrenia with an isolated and an explained deficiency of vitamin K dependent factors. The particularity of this intoxication lies in the dangerous and prolonged side effects making the curative treatment difficult and long.

**Disclosure:**

No significant relationships.

